# Supplementary material for: Cholesterol Modulation Attenuates the AD-like Phenotype Induced by Herpes Simplex Virus Type 1 Infection
Source: Biomolecules. 2024 May 20;14(5):603. doi: 10.3390/biom14050603 (PMC11117519; doi:10.3390/biom14050603)
Supplement: Supplementary file 1 [file biomolecules-14-00603-s001.zip › biomolecules-2990345-supplementary.pdf]

Original blots of Figure 3A

Blots ICP4

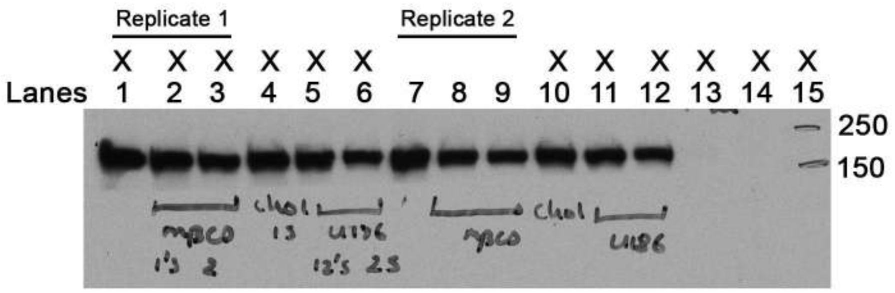

ICP4: replicates 1 and 2

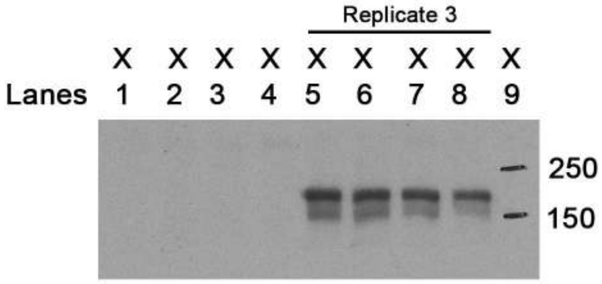

ICP4: replicate 3

Blots gC

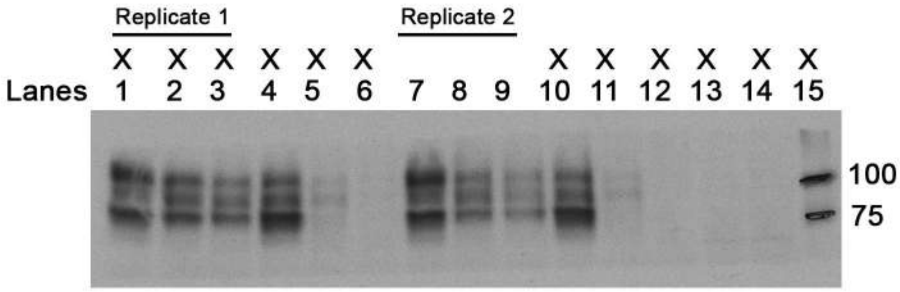

gC: replicates 1 and 2

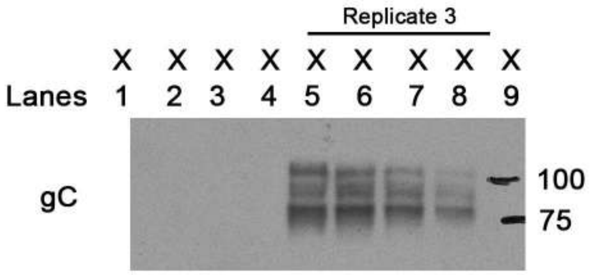

gC: replicate 3

Blots tubulin

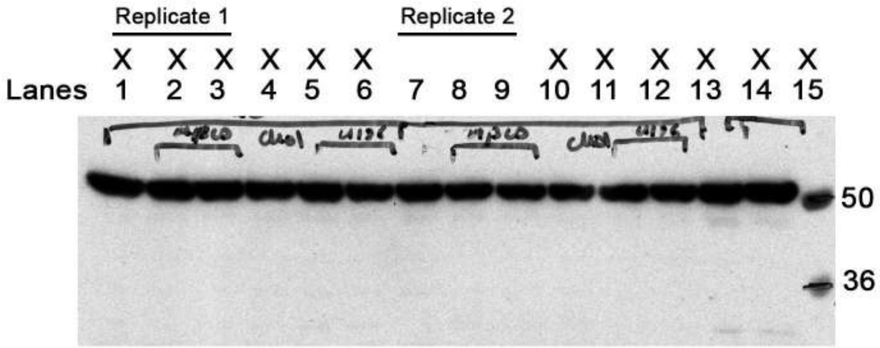

tubulin: replicates 1 and 2

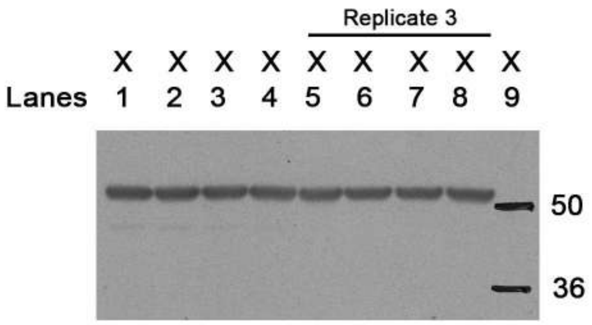

tubulin: replicate 3

Lane	Sample
1	SK-N-MC infected cells moi 10 18 hpi. Exp 1
2	SK-N-MC infected cells moi 10 18 hpi + MBCD 1.5 mM. Exp 1
3	SK-N-MC infected cells moi 10 18 hpi + MBCD 2 mM. Exp 1
4	SK-N-MC infected cells moi 10 18 hpi + Cholesterol 15 µg/ml. Exp 1
5	SK-N-MC infected cells moi 10 18 hpi + U18666A 12.5 µg/ml. Exp 1
6	SK-N-MC infected cells moi 10 18 hpi + U18666A 25 µg/ml. Exp 1
7	SK-N-MC infected cells moi 10 18 hpi. Exp 2
8	SK-N-MC infected cells moi 10 18 hpi + MBCD 1.5 mM. Exp 2
9	SK-N-MC infected cells moi 10 18 hpi + MBCD 2 mM. Exp 2
10	SK-N-MC infected cells moi 10 18 hpi + Cholesterol 15 µg/ml. Exp 2
11	SK-N-MC infected cells moi 10 18 hpi + U18666A 12.5 µg/ml. Exp 2
12	SK-N-MC infected cells moi 10 18 hpi + U18666A 25 µg/ml. Exp 2
13	SK-N-MC mock cells Exp 1
14	SK-N-MC mock cells Exp 2
15	Molecular weight markers<br>(Precision Plus Protein Standards Dual Color (BioRad)

Lane	Sample
1	SK-N-MC mock cells. Exp 3
2	SK-N-MC mock cells + MBCD 1 mM. Exp 3
3	SK-N-MC mock cells + MBCD 1.5 mM. Exp 3
4	SK-N-MC mock cells + MBCD 2 mM. Exp 3
5	SK-N-MC infected cells moi 10 18 hpi. Exp 3
6	SK-N-MC infected cells moi 10 18 hpi + MBCD 1 mM. Exp 3
7	SK-N-MC infected cells moi 10 18 hpi + MBCD 1.5 mM. Exp 3
8	SK-N-MC infected cells moi 10 18 hpi + MBCD 2 mM. Exp 3
9	Molecular weight markers<br>(Precision Plus Protein Standards Dual Color (BioRad)

Original blots of Figure 3B

Blots ICP4

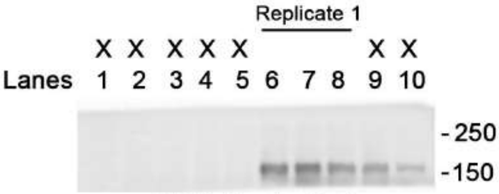

ICP4: Replicate 1

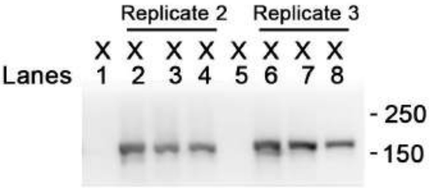

ICP4: Replicate 2 and 3

Blots tubulin of ICP4

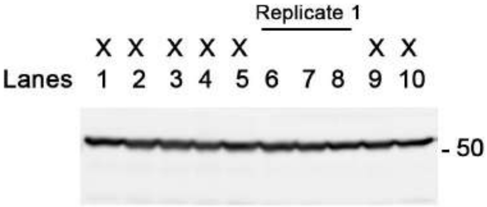

tubulin: Replicate 1

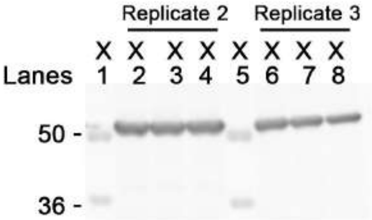

tubulin: Replicate 2 and 3

Lane	Sample
1	N2a mock cells Exp 1
2	N2a mock cells + MBCD 2.5 mM Exp 1
3	N2a mock cells + MBCD 3 mM Exp 1
4	N2a mock cells + MBCD 3.5 mM Exp 1
5	N2a mock cells + MBCD 4 mM Exp 1
6	N2a infected cells moi 10 18 hpi Exp 1
7	N2a infected cells moi 10 18 hpi + MBCD 2.5 mM Exp 1
8	N2a infected cells moi 10 18 hpi + MBCD 3 mM Exp 1
9	N2a infected cells moi 10 18 hpi + MBCD 3.5 mM Exp 1
10	N2a infected cells moi 10 18 hpi + MBCD 4 mM Exp 1

Lane	Sample
1	Molecular weight markers (kDa)<br>(Precision Plus Protein Standards Dual Color (BioRad)
2	N2a infected cells moi 10 18 hpi Exp 2
3	N2a infected cells moi 10 18 hpi + MBCD 2.5 mM Exp 2
4	N2a infected cells moi 10 18 hpi + MBCD 3 mM Exp 2
5	Molecular weight markers (kDa)<br>(Precision Plus Protein Standards Dual Color (BioRad)
6	N2a infected cells moi 10 18 hpi Exp 3
7	N2a infected cells moi 10 18 hpi + MBCD 2.5 mM Exp 3
8	N2a infected cells moi 10 18 hpi + MBCD 3 mM Exp 3

Blots gC

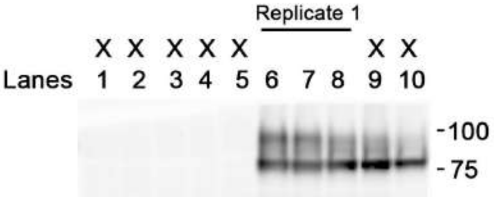

gC: Replicate 1

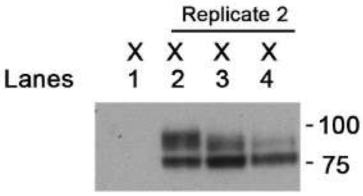

gC: Replicate 2

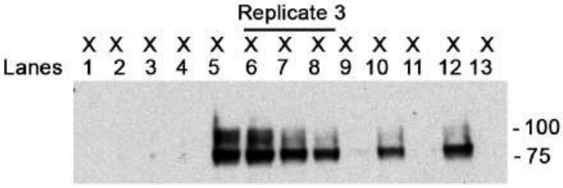

gC: Replicate 3

Blots tubulin of gC

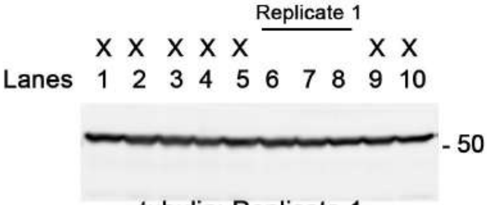

tubulin: Replicate 1

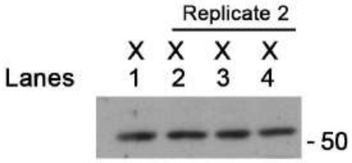

tubulin: Replicate 2

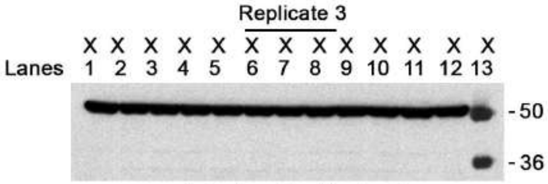

tubulin: Replicate 3

Lane	Sample
1	N2a mock cells Exp 1
2	N2a mock cells + MBCD 2.5 mM Exp 1
3	N2a mock cells + MBCD 3 mM Exp 1
4	N2a mock cells + MBCD 3.5 mM Exp 1
5	N2a mock cells + MBCD 4 mM Exp 1
6	N2a infected cells moi 10 18 hpi Exp 1
7	N2a infected cells moi 10 18 hpi + MBCD 2.5 mM Exp 1
8	N2a infected cells moi 10 18 hpi + MBCD 3 mM Exp 1
9	N2a infected cells moi 10 18 hpi + MBCD 3.5 mM Exp 1
10	N2a infected cells moi 10 18 hpi + MBCD 4 mM Exp 1

Lane	Sample
1	N2a mock cells Exp 2
2	N2a infected cells moi 10 18 hpi Exp 2
3	N2a infected cells moi 10 18 hpi + MBCD 2.5 mM Exp 2
4	N2a infected cells moi 10 18 hpi + MBCD 3 mM Exp 2

Lane	Sample
1	N2a mock cells, control infection
2	N2a mock cells Exp 3
3	N2a mock cells + MBCD 2.5 mM Exp 3
4	N2a mock cells + MBCD 3 mM Exp 3
5	N2a moi 10 cells 18 hpi, control infection
6	N2a infected cells moi 10 18 hpi Exp 3
7	N2a infected cells moi 10 18 hpi + MBCD 2.5 mM Exp 3
8	N2a infected cells moi 10 18 hpi + MBCD 3 mM Exp 3
9	N2a infected cells mock + U18666A 25 μg/ml. Exp 3
10	N2a infected cells moi 10 18 hpi + U18666A 25 μg/ml Exp 3
11	N2a infected cells mock + Cholesterol 15 μg/ml. Exp 3
12	N2a infected cells moi 10 18 hpi + Cholesterol 15 μg/ml Exp 3
13	Molecular weight markers<br>(Precision Plus Protein Standards Dual Color (BioRad)

Original blots of Figure 4A

Blots ICP4

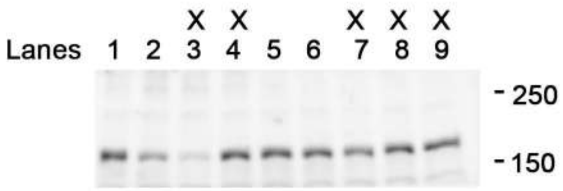

Blot SK-N-MC cells

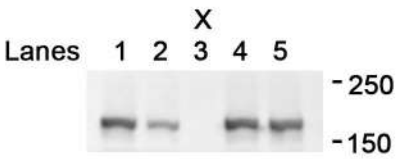

Blot N2a cells

Blots tubulin

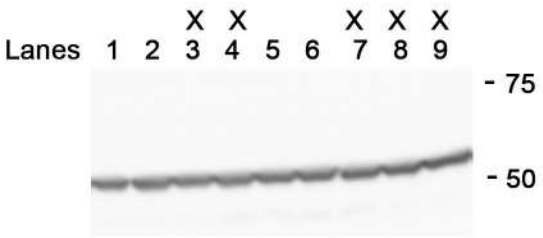

Blot SK-N-MC cells

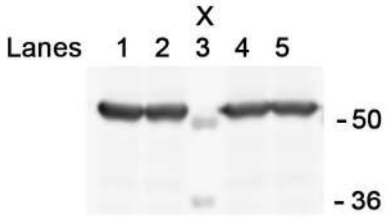

Blot N2a cells

Lane	Sample
1	SK-N-MC infected cells moi 10 5 hpi (-1 hpi)
2	SK-N-MC infected cells moi 10 5 hpi + MBCD 2 mM (-1 hpi)
3	SK-N-MC infected cells moi 10 5 hpi (-1 hpi) + U18666A 12.5 µg/ml
4	SK-N-MC infected cells moi 10 5 hpi (-1 hpi) + Cholesterol 15 µg/ml
5	SK-N-MC infected cells moi 10 5 hpi (0 hpi)
6	SK-N-MC infected cells moi 10 5 hpi + MBCD 2 mM (0 hpi)
7	SK-N-MC infected cells moi 10 5 hpi (0 hpi) + U18666A 12.5 µg/ml
8	SK-N-MC infected cells moi 10 5 hpi (0 hpi) + Cholesterol 15 µg/ml
9	SK-N-MC infected cells moi 10 5 hpi (0 hpi) + PAA 100 µg/ml (viral replication inhibitor)

Lane	Sample
1	N2a infected cells moi 10 5 hpi (-1 hpi)
2	N2a infected cells moi 10 5 hpi + MBCD 3 mM (-1 hpi)
3	Molecular weight markers (kDa)<br>(Precision Plus Protein Standards Dual Color (BioRad)
4	N2a infected cells moi 10 5 hpi (0 hpi)
5	N2a infected cells moi 10 5 hpi + MBCD 3 mM (0 hpi)

Original blots of Figure 4C

Blots ICP4

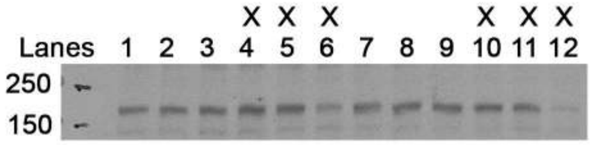

Blot SK-N-MC cells

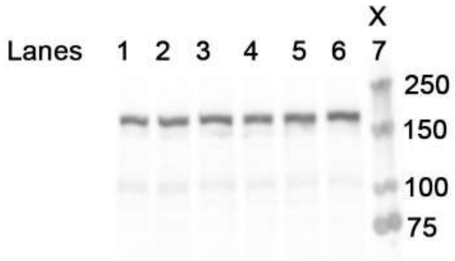

Blot N2a cells

Blots tubulin

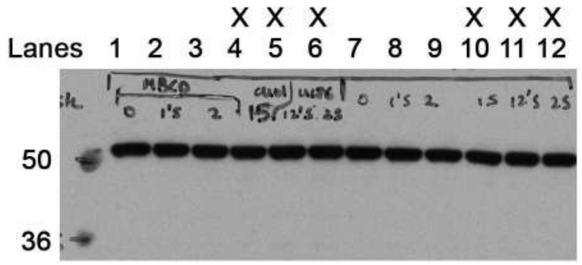

Blot SK-N-MC cells

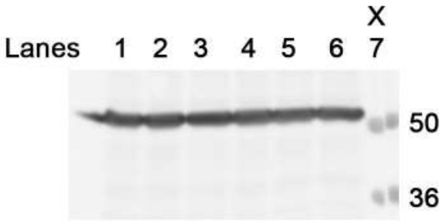

Blot N2a cells

Lane	Sample
1	SK-N-MC infected cells moi 10 5 hpi
2	SK-N-MC infected cells moi 10 5 hpi + MBCD 1.5 mM
3	SK-N-MC infected cells moi 10 5 hpi + MBCD 2 mM
4	SK-N-MC infected cells moi 10 5 hpi + Cholesterol 15 μg/ml
5	SK-N-MC infected cells cells moi 5 18 hpi + U18666A 12.5 μg/ml
6	SK-N-MC infected cells cells moi 5 18 hpi + U18666A 25 μg/ml
7	SK-N-MC infected cells moi 10 5 hpi + citrate
8	SK-N-MC infected cells moi 10 5 hpi + citrate + MBCD 1.5 mM
9	SK-N-MC infected cells moi 10 5 hpi + citrate + MBCD 2 mM
10	SK-N-MC infected cells moi 10 5 hpi + citrate + Cholesterol 15 μg/ml
11	SK-N-MC infected cells cells moi 10 5 hpi + citrate + U18666A 12.5 μg/ml
12	SK-N-MC infected cells cells moi 10 5 hpi + citrate + U18666A 25 μg/ml

Lane	Sample
1	N2a infected cells moi 10 5 hpi
2	N2a infected cells moi 10 5 hpi + MBCD 2.5 mM
3	N2a infected cells moi 10 5 hpi + MBCD 3 mM
4	N2a infected cells moi 10 5 hpi + citrate
5	N2a infected cells moi 10 5 hpi + citrate + MBCD 2.5 mM
6	N2a infected cells moi 10 5 hpi + citrate + MBCD 3 mM
7	Molecular weight markers (kDa)<br>(Precision Plus Protein Standards Dual Color (BioRad)

Original blots of Figure 4D

Blots gC

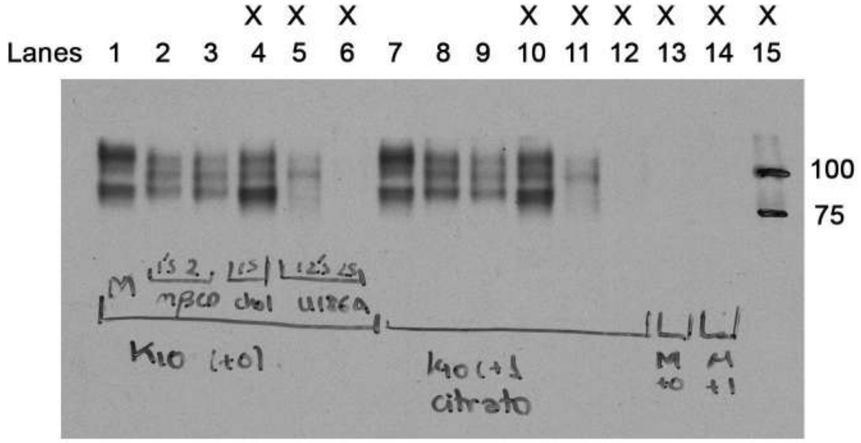

blot SK-N-MC cells

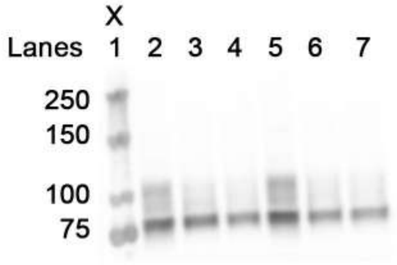

blot N2a cells

Blots tubulin

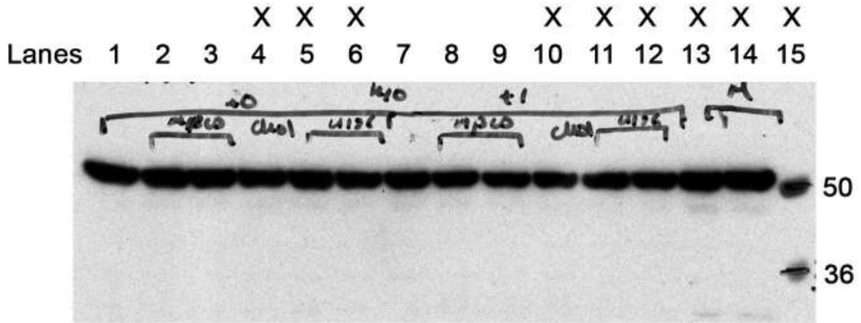

blot SK-N-MC cells

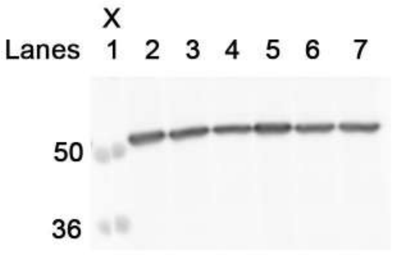

blot N2a cells

Lane	Sample
1	SK-N-MC infected cells moi 10 18 hpi
2	SK-N-MC infected cells moi 10 18 hpi + MBCD 1.5 mM
3	SK-N-MC infected cells moi 10 18 hpi + MBCD 2 mM
4	SK-N-MC infected cells moi 10 18 hpi + Cholesterol 15 μg/ml
5	SK-N-MC infected cells cells moi 10 18 hpi + U18666A 12.5 μg/ml
6	SK-N-MC infected cells cells moi 10 18 hpi + U18666A 25 μg/ml
7	SK-N-MC infected cells moi 10 18 hpi + citrate
8	SK-N-MC infected cells moi 10 18 hpi + citrate + MBCD 1.5 mM
9	SK-N-MC infected cells moi 10 18 hpi + citrate + MBCD 2 mM
10	SK-N-MC infected cells moi 10 18 hpi + citrate + Cholesterol 15 μg/ml
11	SK-N-MC infected cells cells moi 10 18 hpi + citrate + U18666A 12.5 μg/ml
12	SK-N-MC infected cells cells moi 10 18 hpi + citrate + U18666A 25 μg/ml
13	SK-N-MC mock cells
14	SK-N-MC mock cells + citrate
15	Molecular weight markers (kDa)<br>(Precision Plus Protein Standards Dual Color (BioRad)

Lane	Sample
1	Molecular weight markers (kDa)<br>(Precision Plus Protein Standards Dual Color (BioRad)
2	N2a infected cells moi 10 18 hpi
3	N2a infected cells moi 10 18 hpi + MBCD 2.5 mM
4	N2a infected cells moi 10 18 hpi + MBCD 3 mM
5	N2a infected cells moi 10 18 hpi + citrate
6	N2a infected cells moi 10 18 hpi + citrate + MBCD 2.5 mM
7	N2a infected cells moi 10 18 hpi + citrate + MBCD 3 mM

Original blots of Figure 6F

Blot Ser422

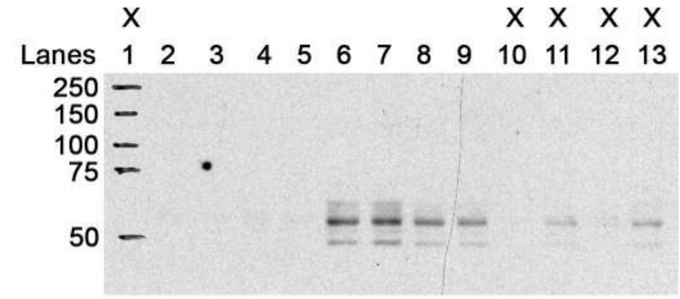

Blot tubulin

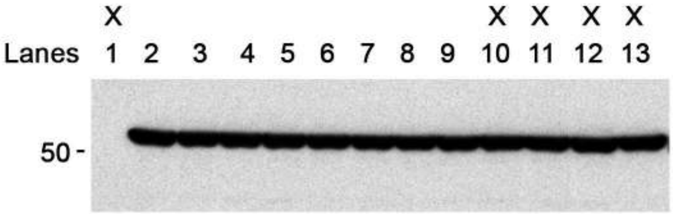

Lane	Sample
1	Molecular weight markers (kDa)<br>(Precision Plus Protein Standards Dual Color (BioRad)
2	SK-N-MC mock cells 18 hpi
3	SK-N-MC mock cells 18 hpi + MBCD 1 mM
4	SK-N-MC mock cells 18 hpi + MBCD 1.5 mM
5	SK-N-MC mock cells 18 hpi + MBCD 2 mM
6	SK-N-MC infected cells moi 10 18 hpi
7	SK-N-MC infected cells moi 10 18 hpi + MBCD 1 mM
8	SK-N-MC infected cells moi 10 18 hpi + MBCD 1.5 mM
9	SK-N-MC infected cells moi 10 18 hpi + MBCD 2 mM
10	SK-N-MC mock cells + U18666A 25 μg/ml
11	SK-N-MC infected cells moi 10 18 hpi + U18666A 25 μg/ml
12	SK-N-MC mock cells + Cholesterol 15 μg/ml
13	SK-N-MC infected cells moi 10 18 hpi + Cholesterol 15 μg/ml
